# Statistical Epistasis and Functional Brain Imaging Support a Role of Voltage-Gated Potassium Channels in Human Memory

**DOI:** 10.1371/journal.pone.0029337

**Published:** 2011-12-21

**Authors:** Angela Heck, Christian Vogler, Leo Gschwind, Sandra Ackermann, Bianca Auschra, Klara Spalek, Björn Rasch, Dominique de Quervain, Andreas Papassotiropoulos

**Affiliations:** 1 Division of Molecular Neuroscience, Department of Psychology, University of Basel, Basel, Switzerland; 2 Division of Molecular Neuroscience, University Psychiatric Clinics, University of Basel, Basel, Switzerland; 3 Life Sciences Training Facility, Department Biozentrum, University of Basel, Basel, Switzerland; 4 Division of Cognitive Neuroscience, Department of Psychology, University of Basel, Basel, Switzerland; 5 Division of Cognitive Neuroscience, University Psychiatric Clinics, University of Basel, Basel, Switzerland; University of Turin, Italy

## Abstract

Despite the current progress in high-throughput, dense genome scans, a major portion of complex traits' heritability still remains unexplained, a phenomenon commonly termed “missing heritability.” The negligence of analytical approaches accounting for gene-gene interaction effects, such as statistical epistasis, is probably central to this phenomenon. Here we performed a comprehensive two-way SNP interaction analysis of human episodic memory, which is a heritable complex trait, and focused on 120 genes known to show differential, memory-related expression patterns in rat hippocampus. Functional magnetic resonance imaging was also used to capture genotype-dependent differences in memory-related brain activity. A significant, episodic memory-related interaction between two markers located in potassium channel genes (*KCNB2* and *KCNH5*) was observed (*P*_nominal combined_ = 0.000001). The epistatic interaction was robust, as it was significant in a screening (*P*_nominal_ = 0.0000012) and in a replication sample (*P*_nominal_ = 0.01). Finally, we found genotype-dependent activity differences in the parahippocampal gyrus (*P*_nominal_ = 0.001) supporting the behavioral genetics finding. Our results demonstrate the importance of analytical approaches that go beyond single marker statistics of complex traits.

## Introduction

Unbiased genome-wide association studies (GWAS) may powerfully identify components of the genetic basis of complex, multigenic traits. In the last 5 years, GWAS using hundreds of thousands of polymorphic markers, mainly single nucleotide polymorphisms (SNPs), in samples ranging from a few hundreds to several thousands of individuals have led and still lead to the identification of numerous susceptibility genes and trait-related genomic variants. However, despite this extensive use of genetic and analytical force, which has undoubtedly proven successful to disentangle additive genetic effects, the portion of the complex traits' heritability related to non-additive effects remains largely unexplained. This contributes to the phenomenon, commonly termed “missing heritability [1]. Therefore, in addition to parent-of-origin effects and epigenetic factors, causal but non-examined rare variants, and environmental influences, the negligence of analytical approaches accounting for gene-gene interaction effects, such as statistical epistasis, is probably central to this phenomenon. Indeed, despite the obvious conception that the analysis of genetically complex traits should account for the underlying biological and statistical complexity, the vast majority of large-scale genetic association studies to date are restricted to the use of single marker statistics. Clearly, this approach does not fully account for the polygenic nature of the phenotype under study and erroneously implies that the impact of genetic variation is comparable to a major gene effect.

The above also holds true for the study of the genetics of episodic memory, which is the ability to encode and retrieve a particular event along with its contextual information. Episodic memory is a polygenic trait, characterized by large interindividual variability and substantial heritability estimates [2], [3]. Although the large-scale analysis of statistical epistasis related to complex traits has proven feasible [4], only few studies have addressed epistatic effects of a limited number of genetic variants on cognitive functions [5], [6]. However, studies involving comprehensive sets of genetic variants and addressing epistatic effects on human memory are lacking.

The concept of statistical epistasis was defined about a century ago and deals with the statistical deviation from additive interaction effects between genetic markers [7]. Per definition, the inclusion of such statistical interaction terms as epistasis exponentially increases the number of statistical tests performed. For example, a two-way interaction analysis between 1 000 single markers requires the performance of 499 500 tests. Some strategies attempting to limit the number of necessary tests in analyses of epistasis employ a stepwise procedure by including only those interaction terms, for which the corresponding marker showed a significant main effect in the first step single-marker analysis. However, this approach is arbitrary as there is no biological rationale for considering only markers with significant main effects. Indeed, a recently published report of an exhaustive genome-wide analysis showed that significant epistatic interactions would have been missed if SNPs that did not display any main effect had been excluded *a priori* [8].

Here we performed a comprehensive two-way SNP interaction analysis of human episodic memory and included genetic markers irrespective of their main effects. In order to counteract the inflation of type I error caused by the exponential increase of statistical tests we focused on 120 genes known to show differential, time-dependent expression patterns in the hippocampus of rats performing a Morris water maze task [9]. In addition, we used functional magnetic resonance imaging (fMRI) to capture genotype-dependent effects on brain activity underlying episodic memory to validate the genetic findings.

We show that the interaction of two markers located in potassium channel genes (*KCNB2* and *KCNH5*) was significantly associated with episodic memory performance. The epistatic interaction was successfully replicated in a second, independent sample. Interestingly, the SNPs forming the interaction term would have been missed in a single marker analysis. Finally, we found genotype-dependent activity differences in the parahippocampal gyrus in an episodic memory task supporting the behavioral genetics finding.

## Results

### SNP-SNP interactions and episodic memory performance

**Figure 1:**
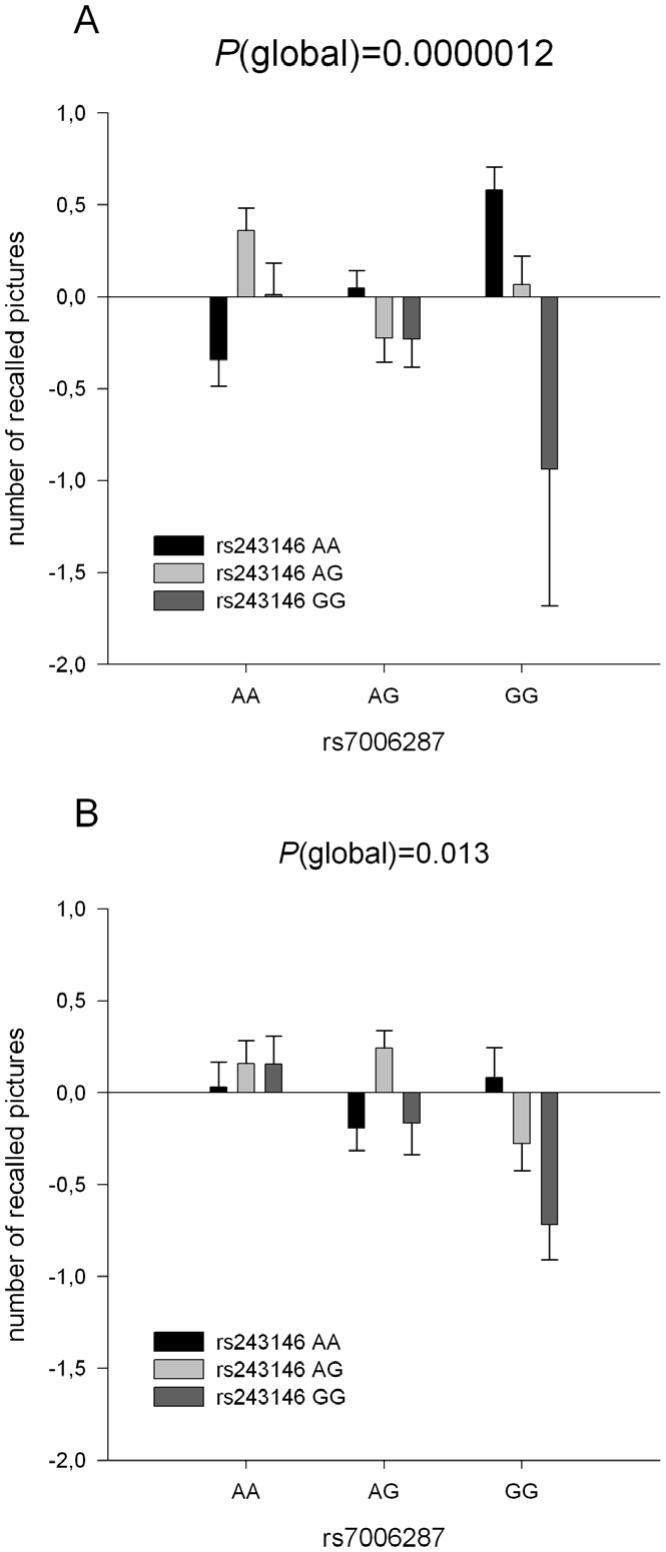
Number of recalled pictures (z-transformed means and standard error of the mean) as a function of rs243146 (A/G) genotype and rs7006287 (A/G) genotype (A) in the screening sample (B) in the replication sample.

Based on 485 SNPs, a total of 117 370 valid tests of pairwise interactions were performed. Of these, two interaction terms reached a corrected p-value *P*_FDR_<0.1 and were subsequently studied in the replication sample. Notably, both interaction terms include SNPs mapping on the gene coding for the potassium voltage-gated channel, subfamily H (*KCNH5*), and the gene encoding the shab-related potassium voltage-gated channel (*KCNB2*). From the SNP pairs analyzed in the replication sample (*n* = 2, one-sided testing), the interaction term involving *KCNH5* SNP rs243146 and *KCNB2* SNP rs7006287 showed the lowest p-value (*P*_nominal_ = 0.013), with the same direction of effect as in the screening sample (see Table 1; *P*_nominal combined_ = 0.000001). In both samples, the interaction between SNP rs243146 and SNP rs7006287 revealed a beneficial effect of major allele load of SNP rs243146 on memory performance among GG-carriers, but not among AG- and AA-carriers of SNP rs7006287 (see Figure 1A and 1B). To clarify the direction of effect, we performed post-hoc tests in both samples. In the screening sample, subjects homozygous for the G-allele of SNP rs7006287 showed an additive effect for the major allele of rs243146 (*ß* = −0.39, *P* = 0.00024), and this effect was reverted in AA-carriers of SNP rs7006287 (*ß* = 0.24, *P* = 0.015). No effect of rs243146 could be observed in the heterozygous AG-carriers (*ß* = −0.12, *P* = 0.12). A very similar pattern was observed in the replication sample: Whereas subjects homozygous for the G-allele of SNP rs7006287 again showed an additive effect for the major allele of rs243146 (*ß* = −0.32, *P* = 0.003), we observed no such effect in the AA and the AG-carriers of SNP rs7006287 (*ß* = 0.06, *P* = 0.53 and *ß* = 0.09, *P* = 0.22, respectively).

**Table 1:** Performance in the picture-based episodic memory task according to genotype in the screening and in the replication sample.

Screening sample (*n* = 359)												
*KCNB2* (rs7006287) - *KCNH5* (rs243146) genotypes												
	AA/AAn = 43	AA/AGn = 49	AA/GGn = 14	AG/AAn = 63	AG/AGn = 72	AG/GGn = 33	GG/AAn = 39	GG/AGn = 35	GG/GG n = 9	Beta	Stat	p-value
Number of recalled pictures (mean, s.e.)	15.98±0.53	18.57±0.46	17.29±0.64	17.41±0.36	16.42±0.84	16.39±0.56	19.38±0.46	17.49±0.58	13.78±2.73	−1.83	23.55	0.0000012

Replication sample (*n* = 422)												
*KCNB2* (rs7006287) - *KCNH5* (rs243146) genotypes												
	AA/AAn = 56	AA/AGn = 68	AA/GGn = 11	AG/AAn = 80	AG/AGn = 97	AG/GGn = 27	GG/AAn = 35	GG/AGn = 31	GG/GG n = 16	Beta	Stat	p-value
Number of recalled pictures (mean, s.e.)	29.39±1.17	30.47±1.11	30.45±1.30	27.49±1.03	31.22±0.82	27.74±1.48	29.82±1.41	26.77±1.26	23.00±1.65	−1.89	4.92	0.013

Abbreviations: standard error of the mean, s.e.

Next, we tested in a single marker analysis if the interacting SNPs rs243146 and rs7006287 show main effects for the phenotype of interest in the screening sample. No main effect emerged for rs7006287 (*P*>0.05). SNP rs243146 showed a weak nominal additive effect for association with episodic memory (*P*_uncorrected_ = 0.03). Therefore, by using a non-exhaustive analysis of interaction that is restricted to markers displaying a main effect, the interaction between rs7006287 and rs243146 would have been missed. Across genotype groups, there were no significant differences in potentially confounding variables (age: *F* = 0.21; *P* = 0.96; gender: *χ2* = 8.76; *P* = 0.12). We observed a significant gender effect on episodic memory performance (females performed better than males, p = 0.0001). Nevertheless, the inclusion of gender as covariate in the regression analysis did not alter the results.

Furthermore, we did not observe effects of the interaction term on measures of attention and working memory (*ß* = −0.91; *P* = 0.36 and *ß* = −0.02; *P* = 0.91, respectively).

### KCNB2×KCNH5 interaction is linked to human brain function

**Figure 2:**
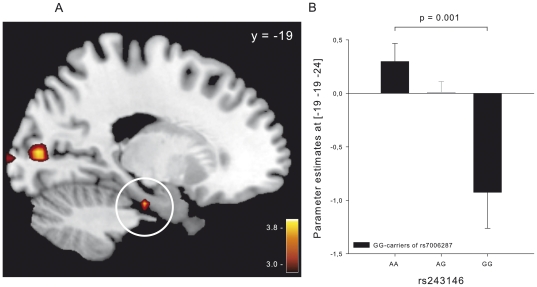
SNP rs243146 genotype-dependent differences in brain activity in the parahippocampal gyrus of rs7006287 GG-carriers during memory encoding. (A) Gene dose-dependent (with increasing numbers of A alleles) increases in activity in the left parahippocampal gyrus (*P* = 0.001, displayed at an uncorrected threshold of *P* = 0.005). The white circle indicates the activation in the left parahippocampal gyrus. (B) Parameter estimates at the peak voxel in the left parahippocampal gyrus (−19, −19, −24).

The descriptive analysis of behavioral data in the fMRI experiment showed the same direction of effect as observed in the screening and the replication sample: Again, in the group of homozygous GG-carriers of *KCNB2* SNP rs7006287, memory performance showed a linear increase, depending on the load of the major (A) allele of *KCNH5* SNP rs243146 (GG (*n* = 3): 27.67±7.97; AG (*n* = 11): 27.55±2.72; AA (*n* = 9): 33.89±3.11). Due to lack in power, the global interaction p-value for rs243146×rs7006287 did not reach statistical significance (*P* = 0.3). Next, we investigated in a genotype-dependent analysis whether brain activation levels in GG-carriers of SNP rs7006287 show an allele-dose effect of the major allele of SNP rs243146. We observed a significant positive correlation in the left parahippocampal gyrus (maximum at [−19,−19,−24] *t* = 3.70, *P* = 0.001, Figure 2). In addition to the genotype-dependent activation in the parahippocampal gyrus, exploratory whole brain analysis showed positive correlations in the superior temporal gyrus, in the inferior and the medial frontal gyrus and in the medial occipital gyrus in response to the picture stimuli (see Table 2). The genotype-independent analysis revealed that the memory task led to robust activations in the left parahippocampal gyrus and in the right hippocampus (maximum at [−22,−11,−20] *t* = 13.14, *P*<0.001 and [22,−8,−24] *t* = 14.38, *P*<0.001, respectively). The task-related parahippocampal activation was also significant at the same voxel where maximal genotype-dependent activation difference was observed ([−19,−19,−24] *t* = 7.43, *P*<0.001). No brain region showed activation for the inverse contrast (i.e. scrambled compared to all pictures). Our finding is in line with studies repeatedly reporting parahippocampal activation during encoding of picture stimuli and that the parahippocampal gyrus is particularly involved in the memory-related processing of the spatial context of a certain event [10]–[12].

**Table 2:** Brain regions significantly associated with the number of A-alleles of SNP rs243146 in the group of GG-carriers of SNP rs7006287.

				MNI coordinates					
Region	BA	Number of voxels	L/R	x	y	z	*t*(1,21)	*Z*	*P*
All>scrambled pictures									
Parahippocampal gyrus	28	2	L	−19	−19	−24	3.70	3.21	0.001
Superior temporal gyrus	38	20	L	−50	11	−20	4.98	4.00	<0.001
	42	2	L	−63	−25	12	3.73	3.23	0.001
Medial frontal gyrus	6	7	R	36	8	52	4.62	3.79	<0.001
	8	6	R	44	19	48	4.06	3.45	<0.001
Medial occipital gyrus	18	7	L	−30	−88	0	4.28	3.59	<0.001
Inferior frontal gyrus	47	4	R	47	22	−12	3.94	3.37	<0.001
Cuneus	17	7	L	−16	−85	4	3.80	3.28	0.001
Scrambled>all pictures									

No suprathreshold clusters.

BA: Brodmann's area; L/R: left/right hemisphere.

## Discussion

We provide first evidence for an epistatic effect of polymorphisms mapping on the potassium voltage-gated ion channels *KCNB2* and *KCNH5* on episodic memory performance. By searching for two-way interactions in a panel of candidate genes implicated in memory, we observed an additive interaction between *KCNB2* SNP rs7006287 and *KCNH5* SNP rs243146. This effect was replicated in a second sample, i.e. the identical SNP pair showed the same significant direction of effect. Post-hoc analyses revealed that the interaction is based on the moderating role of *KCNB2* SNP rs7006287: Whereas in the carriers of both G-alleles of SNP rs7006287 memory performance is moderated by the number of A-alleles of SNP rs243146, no such moderating effect could be observed in the AG- and AA-allele carriers of rs7006287. Additionally, we observed the same pattern in an fMRI experiment during picture encoding: In GG-carriers of rs7006287, an increase of BOLD signal according to the gene-dose of the A-allele of SNP rs243149 was observed in the parahippocampal region. Notably, several findings of non-linear interactions on hippocampal function during memory performance (e.g recognition and working memory) have been reported in the literature [13]–[15].

In the screening sample, the effect size (ES) for the interaction term was small to moderate (*f* = 0.25) [16], which is in line with prior reports of epistatic effects on cognitive functions [5], [6]. Our sample size was sufficient to detect at least medium ES (*f* = 0.30) with a power of 80% (error probability *α* = 0.000001). The statistical power of the replication sample for detecting ES similar to those observed in the screening sample at *α* = 0.025 (i.e. corrected for two interaction terms) was 99%. It is widely acknowledged that the ES in imaging genetic studies investigating memory functions significantly exceed the usually observed ES in behavioral genetic association studies [17]–[20]. Also in this study, the ES in the brain imaging experiment was large (f = 0.82). Post-hoc power analysis revealed that our fMRI sample had a power of 99% to detect the interaction effect with an error probability of *α* = 0.001.

Within the large group of potassium-selective channels, the proteins encoded by *KCNB2* (NM_004770, chr8q13.2) and *KCNH5* (NM_139318, chr14q23.1) belong to the family of voltage-gated potassium channels (K_V_). K_V_ play a pivotal role in cell excitability, regulation of neuronal firing and action potential form, and they are also implicated as key players for synaptic plasticity [21]. KCNB2 (K_v_2.2) is a delayed rectifier potassium channel and an important modulator of somatodendritic excitability [22]. Whereas it is well known that the second shab-related delayed rectifier potassium channel (K_v_2.1) is expressed in most parts of the mammalian brain [23], [24], the expression pattern for K_v_2.2 is less clear. Nevertheless, K_v_2.2 expression has been reported in the somata and dendrites of rat hippocampal neurons [25], [26]. Furthermore, in rats with scopolamine-induced cognitive impairment, an increased peak amplitude and current density of K_v_2.1 could be detected in isolated hippocampal pyramidal neurons, an effect which is potentially mediated by a parallel increase of mRNA expression in pyramidal hippocampal cells [27]. Human genetic association studies investigating *KCNB2* are rare: Besides a possible SNP-SNP interaction for the risk of migraine (*KCNB2* SNP rs1431656 and a SNP mapping on the beta 2 subunit of the calcium dependent voltage channel; *CACNB2*, [28]), a GWAS identified *KCNB2* as candidate gene for a risk factor in cardiovascular disease [29].

KCNH5 (K_v_10.2) is a subunit of a pore-forming outward rectifying potassium channel that belongs to the EAG (ether-a-go-go) subfamily of voltage-gated potassium channels. EAG channels are characterized by activation in lower voltage range as compared to the closely related K_v_10.1 channel (KCNH1) [30]. Its expression pattern is restricted to the nervous system, and expression in rat hippocampal tissue has been reported [31], [32]. In *Drosophila melanogaster*, mutants of the EAG-related potassium channel show changes in neuronal plasticity, associative learning and habituation [33], [34]. Up to now, besides a genome-wide hit for antipsychotic treatment [35], no genetic association study exists that investigates if *KCNH5* relates to human cognitive functions. Notably, both SNPs rs7006287 and rs243146 are intronic and with no clear influenced by gene expression. Therefore resequencing should be used to identify the causal variants in linkage disequilibrium with these common SNPs.

It is important to stress some limitations of the present study: Firstly, even though we aimed at analyzing a comprehensive gene list, the SNP panel is restricted to those polymorphisms covered by the Affymetrix 6.0 SNP array. Thus, potentially important gene-gene interactions might have been missed. Moreover, the behavioral genetics approach reported herein only partially captures changes related to gene expression, because of the existence of genetic variations – independent factors, such as epigenetic changes - which also influence gene expression [36]–[38]. Secondly, the analysis of epistatic interactions in large groups of candidate genes results in a high burden of multiple testing. The approach proposed herein reflects a necessary compromise between coverage of genetic variability and the restriction of multiple testing. Thirdly, a MAF set at 35% may be rather stringent. However, this threshold prevents from too sparse cell frequencies when combining genotypes for two-way interaction terms. Fourthly, we restricted our statistical analysis to the use of the additive genetic model to prevent further inflation of type I error. However, other genetic models (i.e. dominant, recessive) and non-linear interactions (i.e. molecular heterosis) may also apply. Finally, because the effects presented herein are significant on an uncorrected level of significance, the fMRI data have to be treated as preliminary and need further replication.

In summary, the analysis of SNP-SNP interactions in a panel of experimentally-driven candidate genes and subsequent replication in an independent sample provides first evidence for interacting effects between *KCNB2* and *KCNH5* on human episodic memory performance. Experimental fMRI data added further support and suggests that the interacting variants are also related to brain activity in the parahippocampal gyrus, a brain structure centrally involved in episodic memory formation. Interestingly, the SNPs included in the interaction term would have been missed in a single marker analysis. Our results indicate, that GWAS focusing on single-marker main effects only are likely to miss genetic effects related to interactions in the absence of significant main effects We are fully aware that the chosen approach is just one amongst other statistical and function-based techniques to delineate the polygenic complexity of episodic memory [39]. Further studies are needed to replicate and corroborate these results by investigating epistatic interaction effects on other cognitive domains, and to address also the importance of non-additive and non-linear interactions, which are hitherto rarely studied.

## Materials and Methods

### Screening sample

The screening sample (413 young Swiss adults, 313 females; mean age: 21.2±1.9) was recruited in the city of Zurich, Switzerland. After complete description of the study, all participants gave written informed consent and performed a picture-based episodic memory task [40]. Briefly, subjects were presented 30 picture stimuli (10 positive, 10 neutral, 10 negative) that were taken from the International Affective Picture System (IAPS; [41]). After a delay of 10 minutes, subjects were unexpectedly instructed to freely recall the pictures. The phenotype of interest (i.e. episodic memory) was measured as the total number of recalled pictures. Additionally, concentration and attention were measured with the d2 cancellation test, and working memory was assessed with the digit span test.

Population stratification is a possible cause for spurious associations and for lack of replication of genetic association findings. Therefore, we tested for population stratification in our genetic dataset by applying the EIGENSOFT software, which corrects for population stratification via principal component analysis (PCA) and calculates lambda (λ) as an indicator for admixture [42]. Admixture was low in the screening sample (*λ* = 1.002). Nevertheless, we defined a PCA-based outlier removal criterion (six standard deviations), because epistatic effects are particularly sensitive to even subtle population admixture [43]–[45]. PCA was run with five iterations (default). After this procedure, which led to the final inclusion of 359 subjects, population admixture was even lower (*λ* = 1.001).

### Replication sample

The replication sample (500 young Swiss adults, 329 females; mean age: 22.5±3.6) was recruited in the city of Basel, Switzerland. After complete description of the study, all participants gave written informed consent. Subjects were presented 72 IAPS pictures (24 positive, 24 negative and 24 neutral) and, after a 10 minutes delay, they were asked to freely recall the pictures. The phenotype of interest (i.e. episodic memory) was measured as the total number of recalled pictures.

The replication sample showed moderate admixture (*λ* = 1.065). After PCA-based outlier detection, which led to the final inclusion of 422 subjects, population admixture was absent (*λ* = 1.000).

For both the screening and the replication study the respective cantonal ethics committees (Zurich and Basel) approved the study protocols.

### Candidate genes

We based our search on genes that were differentially expressed in the hippocampal formation of rats at different time points after a Morris water maze task [9]. Availability of the Genbank numbers of the differentially expressed transcripts was verified in the UCSC Genome Browser on Rat Nov. 2004 (Baylor 3.4/rn4) Assembly (http://genome.ucsc.edu/). Based on the Genbank numbers or the RefSeq gene symbol as provided in the Cavallaro et al. study, 135 human orthologues were identified. Xchromosomal genes (n = 5) and duplicate transcripts were excluded (n = 15), leading to a total of 120 genes. All available SNPs mapping on these genes entered the analysis (for details of SNP selection, see next paragraph). Not considering X-chromosomal genes (*n* = 5), 120 autosomal human orthologs were defined, comprising 12 G protein-coupled receptor genes and their effectors, 11 ion channel genes, 13 ligand-gated ion channels, 13 neuropeptides and growth factors including their receptors, 6 intracellular signaling genes, 6 neurotransmitter transporters, 12 signaling enzymes, 5 proteins involved in signal transduction, 13 synaptic proteins, 17 cell-cell interaction and cytoskeletal proteins, 4 apoptosis genes, 3 enzyme genes and 5 genes involved in transcription or translation regulation. Gene symbols, RefSeq accession numbers and chromosomal locations according to NCBI Build 36.1/hg18 are reported in Table S1, Supplementary Material.

### SNP selection

As a first step, 5 200 intragenic SNPs that are present on the Affymetrix Human SNP-array 6.0 were selected to represent the variability of 108 genes. Next, we applied stringent SNP quality control to gain power for the detection of interaction effects in our samples and to avoid low cell frequencies caused by rare allelic combinations. The following inclusion criteria were set: minor allele frequency (MAF)>35%, non-deviance from Hardy-Weinberg equilibrium (*P*_Fisher_>0.05), call rate>99%, i.e. a SNP was excluded if more than 1% of all individuals failed to have genotypic information for this SNP. This quality control step reduced the number of SNPs suitable for analysis to 1 044. To eliminate redundant information due to strong linkage disequilibrium (LD), we further pruned SNPs in high pairwise LD (*r^2^*>0.85) using the *pruning* option in PLINK, resulting in a final set of 485 SNPs mapping on 71 genes (Table 1, Supplementary Material).

### Array-based SNP genotyping

Samples were processed as described in the Genome-Wide Human SNP Nsp/Sty 6.0 User Guide (Affymetrix). Briefly, genomic DNA concentration was determined by using a Nano-Drop ND-1000 and adjusted to 50 ng/µl in water. 250 ng of DNA was digested in parallel with 10 units of Sty I and Nsp I restriction enzymes (New England Biolabs, Beverly, MA) for 2 hours at 37°C. Enzyme specific adaptor oligonucleotides were then ligated onto the digested ends with T4 DNA Ligase for 3 hours at 16°C. After adjustment to 100 µl with water, 10 µl of the diluted ligation reactions were subjected to PCR. Three PCR reactions of 100 µl were performed for Sty digested products and four PCR reactions for Nsp. PCR was performed with Titanium Taq DNA Polymerase (Clontech, Mountain View, CA) in the presence of 4.5 µM PCR primer 002 (Affymetrix), 350 µM each dNTP (Clontech), 1 M G-C Melt (Clontech), and 1× Titanium Taq PCR Buffer (Clontech). Cycling parameters were as follows: initial denaturation at 94°C for 3 minutes, amplification at 94°C for 30 seconds, 60°C for 45 seconds and extension at 68°C for 15 seconds repeated a total of 30 times, final extension at 68°C for 7 minutes. Reactions were then verified to migrate at an average size between 200–1100 bps using 2% TBE gel electrophoresis. PCR products were combined and purified with the Filter Bottom Plate (Seahorse Bioscience, North Billerica, MA) using Agencourt Magnetic Beads (Beckman Coulter, Fullerton, CA). Purified PCR products were quantified on a Zenith 200 rt microplate reader (Anthos-Labtec, Cambridge, UK). 4 to 5 µg/µl were obtained on average for each sample. From this stage on, the SNP Nsp/Sty 5.0/6.0 Assay Kit (Affymetrix) was used. Around 250 µg of purified PCR products were fragmented using 0.5 units of DNAse I at 37°C for 35 minutes. Fragmentation of the products to an average size less than 180 bps was verified using 4% TBE gel electrophoresis. Following fragmentation, the DNA was end labeled with 105 units of terminal deoxynucleotidyl transferase at 37°C for 4 hours. The labeled DNA was then hybridized onto Genome-Wide Human SNP 6.0 Array at 50°C for 18 hours at 60 rpm. The hybridized array was washed, stained, and scanned according to the manufacturer's (Affymetrix) instructions using Affymetrix GeneChip Command Console (AGCC, version 3.0.1.1214). Generation of SNP calls and Array quality control were performed using the command line programs of the Affymetrix Power Tools package (version: apt-1.12.0). According to the manufacturer's recommendation, Contrast QC was chosen as QC metric, using the default value of greater or equal than 0.4. Mean Call Rate for all samples averaged >98.5%. All samples passing QC criteria were subsequently genotyped using the Birdseed (v2) algorithm.

### fMRI experiment

#### Subjects

In total, 94 young Swiss subjects (60 females; mean age: 23.98±3.97) were recruited for an fMRI study at the University of Zurich and performed a picture memory task [46], [47]. Twenty-three of 94 subjects were homozygous for the G-allele of SNP rs7006287, and within this group, genotype distribution of SNP rs243146 (A,G) was as follows: 9 AA-carriers, 11 AG-carriers and 3 GG-carriers.

In short, after positioned in the scanner, subjects were presented 72 IAPS pictures and 24 scrambled pictures for 2.5 seconds each. During an intertrial period (9–12 seconds) subjects subjectively rated the picture showing scenes according to valence (negative, neutral, positive) and arousal (large, medium, small) on a three-point scale (Self Assessment Manikin, SAM) by pressing a button with a finger of their dominant hand. For scramble pictures, participants rated form (vertical, symmetric or horizontal) and size (large, medium, small) of the geometrical object in the foreground. At ten minutes after picture encoding in the scanner, subjects left the scanner and performed an unexpected delayed free recall. The number of successfully recalled pictures was used as behavioural phenotypic variable.

All subjects were free of any lifetime neurological or psychiatric illness and did not take any medication at the time of the experiment.

The study protocol was approved by the ethics committee of the Canton of Zürich, and written informed consent was obtained from all subjects prior to participation.

#### fMRI data acquisition and processing

Measurements were performed on a Philips Intera 3 T wholebody MR unit equipped with an eight-channel Philips SENSE head coil. Functional time series were acquired with a sensitivity encoded [48] single-shot echo-planar sequence (SENSE-sshEPI). We used the following acquisition parameters: TE (echo time) = 35 ms, FOV (field of view) = 22 cm, acquisition matrix = 80×80, interpolated to 128×128, voxel size: 2.75×2.75×4 mm3, SENSE acceleration factor R = 2.0. Using a midsaggital scout image, 32 contiguous axial slices were placed along the anterior–posterior commissure (AC–PC) plane covering the entire brain with a TR = 3000 ms (θ = 82°). The first two acquisitions were discarded due to T1 saturation effects.

Preprocessing was performed using SPM5 (Statistical Parametric Mapping, Wellcome Department of Cognitive Neurology, London, UK; http://www.fil.ion.ucl.ac.uk/spm/) implemented in Matlab 2008a (The Mathworks Inc., Natick, MA, USA). Volumes were slice-time corrected to the first slice, realigned to the first acquired volume, normalized into standard stereotactic space (template provided by the Montreal Neurological Institute), and smoothed using an 8 mm full-width-at-half-maximum Gaussian kernel. A 128 s cut-off high pass filter was added to the confound partition of the design matrix to account for low-frequency drifts, and a correction for intrinsic autocorrelations was included in the analysis. For each subject, evoked hemodynamic responses to event-types were modeled with a delta (stick) function corresponding to presentation of each stimulus category (negative, positive, neutral and scrambled pictures, respectively) convolved with a canonical hemodynamic response function within the context of a general linear model (GLM). The contrast of interest was computed for every subject on the first level by comparing all photographs (irrespective of emotional quality) versus scrambled pictures. The resulting contrast parameters were then compared between genotype groups in a random effects model using a multiple regression design by coding the number of alleles as weighting covariate. Second-level analyses were performed with SPM8. The threshold of significance was set at an uncorrected level of *P* = 0.001. Parameter estimates from peak voxels were extracted and re-calculated in SPSS with linear regression analyses.

### Statistical analysis

Analyses of SNP-SNP interactions were done with the *epistasis* option implemented in the PLINK software package [49]. For quantitative phenotypes, the *epistasis* option calculates linear regression analysis by including an interaction term for each pair of SNPs, with each SNP coded according to the number of alleles (i.e. major allele = 1; minor allele = 2). The interaction term is included into the regression equation and tested with one degree of freedom (*df* = 1). For the investigation of possible confounder effects, we calculated analysis of variance (ANOVA) for quantitative traits (age) and χ^2^ tests for qualitative traits (gender) to assess differences between genotypic groups. All non-genetic statistical analyses were done with a standard software package (SPSS, release 18.0 SPSS Inc., Chicago, Illinois). P-values of the PLINK analysis were corrected for multiple testing by applying false discovery rate (FDR; [50] that is implemented in the Bioconductor R package multtest [51]). Redundant SNPs were eliminated based on LD measures. Despite this LD pruning, SNP-SNP interactions are still biased by non-independent measures, since each polymorphism is tested repeatedly against all the remaining polymorphisms (i.e. in our case with 485 selected SNPs, each SNP is repeatedly tested 484 times). We therefore set the multiple correction threshold at *P*_FDR_<0.10. Interactions exceeding this significance level were subsequently tested in the replication sample.

To check for possible main effects, we calculated single marker analysis with the WG-Permer software (www.wg-permer.org) and applied permutation-based correction of nominal p-values (10 000 permutations). This procedure is implemented in the WG-Permer software and corrects for the family-wise error rate taking the LD structure between the SNPs into account [52]. As in the epistasis analysis, we applied the additive model of inheritance.

## Supporting Information

Table S1 Functional classes, Gene names, Gene symbols, RefSeq accession numbers and chromosomal location (NCBI Build 36.1/hg18) of selected genes.
(DOC)
