# Supplementary material for: Statistical Epistasis and Functional Brain Imaging Support a Role of Voltage-Gated Potassium Channels in Human Memory
Source: PLoS One. 2011 Dec 21;6(12):e29337. doi: 10.1371/journal.pone.0029337 (PMC3244442; doi:10.1371/journal.pone.0029337)
Supplement: Table S1 — Functional classes, Gene names, Gene symbols, RefSeq accession numbers and chromosomal location (NCBI Build 36.1/hg18) of selected genes. (DOC) [file pone.0029337.s001.doc]

Supplementary Table S1: Functional classes, Gene names, Gene symbols, RefSeq accession numbers and chromosomal location (NCBI Build 36.1/hg18) of selected genes

|  | Gene names | Gene Symbols | Human RefSeq  accession numbers | Chromosomal Region | Number of analyzed SNPs |
| --- | --- | --- | --- | --- | --- |
| G protein-coupled receptors | Gamma-aminobutyric acid (GABA) B receptor, 1 | GABBR1 | NM_021904 | 6p21.31 | 0^b^ |
|  | Gamma-aminobutyric acid (GABA) B receptor, 2 | GABBR2 | NM_005458 | 9q22.1-q22.3 | 21 |
|  | Dopamine receptor D1 | DRD1 | NM_000794 | 5q35.1 | 0^b^ |
|  | Dopamine receptor D4 | DRD4 | NM_000797 | 11p15.5 | 0^a^ |
|  | Adrenergic, alpha-1A-, receptor | ADRA1A | NM_000680 | 8p21.2 | 5 |
|  | Adrenergic, alpha-1D-, receptor | ADRA1D | NM_000678 | 20p13 | 2 |
|  | Tachykinin receptor 1 | TACR1 | NM_001058 | 2p12 | 8 |
|  | Opiate receptor-like 1 | OPRL1 | NM_182647 | 20q13.33 | 1 |
|  | Adenosine A1 receptor | ADORA1 | NM_000674 | 1q32.1 | 1 |
|  | Insulin receptor | INSR | NM_000208 | 19p13.3-p13.2 | 6 |
|  | Insulin | INS | NM_1185098 | 11.p15.5 | 0^a^ |
|  | 5-hydroxytryptamine (serotonin) receptor 6 | HTR6 | NM_000871 | 1p36-p35 | 1 |
| Ion channels | Sodium channel, voltage-gated, type IX, alpha subunit | SCN9A | NM_02977 | 2q24 | 3 |
|  | Potassium voltage-gated channel, shaker-related subfamily, member 5 | KCNA5 | NM_002234 | 12p13 | 0^b^ |
|  | Potassium voltage-gated channel, shaker-related subfamily, member 6 | KCNA6 | NM_002235 | 12p13 | 4 |
|  | Potassium voltage-gated channel, Shab-related subfamily, member 1 | KCNB1 | NM_004975 | 3q42 | 7 |
|  | Potassium voltage-gated channel, Shab-related subfamily, member 2 | KCNB2 | NM_004770 | 8q13.2 | 10 |
|  | Potassium voltage-gated channel, Shal-related subfamily, member 2 | KCND2 | NM_012281 | 7q31 | 0^b^ |
|  | Potassium inwardly-rectifying channel, subfamily J, member 4 | KCNJ4 | NM_152868 | 22q13.1 | 1 |
|  | Potassium inwardly-rectifying channel, subfamily J, member 11 | KCNJ11 | NM_000525 | 11p15.1 | 2 |
|  | Potassium inwardly-rectifying channel, subfamily J, member 16 | KCNJ16 | NM_018658 | 17q24.3 | 1 |
|  | Potassium voltage-gated channel, subfamily H (eag-related), member 5 | KCNH5 | NM_172375 | 14q23.1 | 19 |
|  | Potassium intermediate/small conductance calcium-activated channel, subfamily N, member 2 | KCNN2 | NM_021614 | 5q22.3 | 9 |
| Ligand-gated ion channels | Gamma-aminobutyric acid (GABA) A receptor, alpha 4 | GABRA4 | NM_000809 | 4p12 | 3 |
|  | Gamma-aminobutyric acid (GABA) A receptor, alpha 5 | GABRA5 | NM_000810 | 15q11.2-q12 | 2 |
|  | Gamma-aminobutyric acid (GABA) A receptor, beta 2 | GABRB2 | NM_021911 | 5q34 | 1 |
|  | Gamma-aminobutyric acid (GABA) A receptor, gamma 2 | GABRG2 | NM_198904 | 5q34 | 5 |
|  | Gamma-aminobutyric acid (GABA) A receptor, pi | GABRP | NM_014211 | 5q33-q34 | 2 |
|  | Glutamate receptor, ionotropic, N-methyl D-aspartate 2A | GRIN2A | NM_000833 | 16p13.2 | 30 |
|  | Glutamate receptor, ionotropic, N-methyl D-aspartate 1 | GRIN1 | NM_000832 | 9q34.3 | 1 |
|  | Glutamate receptor, ionotropic, kainate 1 | GRIK1 | NM_175611 | 21q22.1 | 8 |
|  | Glutamate receptor, ionotropic, kainate 2 | GRIK2 | NM_175768 | 6q16.3-q.21 | 14 |
|  | Cholinergic receptor, nicotinic, alpha 3 | CHRNA3 | NM_000743 | 15q24 | 0^b^ |
|  | Cholinergic receptor, nicotinic, beta 1 (muscle) | CHRNB1 | NM_000747 | 17p13.1 | 0^b^ |
|  | Cholinergic receptor, nicotinic, beta 4 | CHRNB4 | NM_000750 | 15q24 | 0^b^ |
|  | 5-hydroxytryptamine (serotonin) receptor 3A | HTR3A | NM_000869 | 11q23.1 | 0^b^ |
| Neuropeptides and growth factors | Fibroblast growth factor 18 | FGF18 | NM_003862 | 5q34 | 0^b^ |
|  | Transforming growth factor, beta receptor III | TGFBR3 | NM_003243 | 1p33-p32 | 11 |
|  | Insulin-like growth factor binding protein 2, 36kDa | IGFBP2 | NM_000597 | 2q33-q34 | 0^b^ |
|  | Interferon, gamma | IFNG | NM_000619 | 12q14 | 1 |
|  | Brain-derived neurotrophic factor | BDNF | NM_170731 | 11p13 | 1 |
|  | Interleukin 1, beta | IL1B | NM_000576 | 2q14 | 1 |
|  | Interleukin 15 | IL15 | NM_172174 | 4q31 | 3 |
|  | Chemokine (C motif) ligand 2 | XCL2 | NM_003175 | 1q24.2 | 0^a^ |
|  | Interleukin 2 receptor, alpha | IL2RA | NM_000417 | 10p15-p14 | 4 |
|  | GDNF family receptor alpha 2 | GFRA2 | NM_1495 | 8p21.3 | 4 |
|  | Corticotropin releasing hormone | CRH | NM_000756 | 8q13 | 0^a^ |
|  | Complement component 8, beta polypeptide | C8B | NM_000066 | 1p32 | 3 |
|  | Complement component 3 | C3 | NM_00064 | 19p13.3-p13.2 | 6 |
| Intracellular signaling | Inositol 1,4,5-triphosphate receptor, type 1 | ITPR1 | NM_002222 | 3p26.1 | 29 |
|  | ATPase, Ca++ transporting, cardiac muscle, slow twitch 2 | ATP2A2 | NM_170665 | 12q24.11 | 0^b^ |
|  | ATPase, Na+/K+ transporting, alpha 1 polypeptide | ATP1A1 | NM_000701 | 1p21 | 0^b^ |
|  | ATPase, Ca++ transporting, plasma membrane 2 | ATP2B2 | NM_001001331 | 3p25.3 | 22 |
|  | Solute carrier family 24 (sodium/potassium/calcium exchanger), member 2 | SLC24A2 | NM_001193288 | 9p22-p13 | 13 |
|  | Frequenin homolog (Drosophila) | FREQ | NM_014286 | 9q34 | 3 |
| Neurotransmitter transporters | Solute carrier family 32 (GABA vesicular transporter), member 1 | SLC32A1 | NM_080552 | 20q11.23 | 0^a^ |
|  | Solute carrier family 1 (glial high affinity glutamate transporter), member 2 | SLC1A2 | NM_004171 | 11p13-p12 | 0^b^ |
|  | Solute carrier family 1 (neuronal/epithelial high affinity glutamate transporter, system Xag), member 1 | SLC1A1 | NM_004170 | 9p24 | 0^b^ |
|  | Solute carrier family 1 (glial high affinity glutamate transporter), member 3 | SLC1A3 | NM_004172 | 5p13 | 0^b^ |
|  | Solute carrier family 6 (neurotransmitter transporter, serotonin), member 4 | SLC6A4 | NM_001045 | 17q11.2 | 0^b^ |
|  | ATP-binding cassette, sub-family B (MDR/TAP), member 6 | ABCB6 | NM_005689 | 2q36 | 0^a^ |
| Signaling enzymes |  | NOS2 | NM_000625 | 17q11.2-q12 | 6 |
|  | Mitogen-activated protein kinase 14 | MAPK14 | NM_139012 | 6p21.3-p21.2 | 1 |
|  | Dual specificity phosphatase 5 | DUSP5 | NM_004419 | 10q25 | 0^b^ |
|  | Phospholipase D1, phosphatidylcholine-specific | PLD1 | NM_002662 | 3q26 | 3 |
|  | Phosphoinositide-3-kinase, regulatory subunit 1 (p85 alpha) | PIK3R1 | NM_181524 | 5q13.1 | 1 |
|  | P21/Cdc42/Rac1-activated kinase 1 (STE20 homolog, yeast) | PAK1 | NM_001128620 | 11q13-q14 | 1 |
|  | Calcium/calmodulin-dependent protein kinase (CaM kinase) II alpha | CAMK2A | NM_171825 | 5q32 | 1 |
|  | Calcium/calmodulin-dependent protein kinase IV | CAMK4 | NM_001744 | 5q21.3 | 10 |
|  | Mitogen-activated protein kinase 9 | MAPK9 | NM_139068 | 5q.35 | 2 |
|  | Janus kinase 1 (a protein tyrosine kinase) | JAK1 | NM_002227 | 1p32.3-p31.3 | 2 |
|  | 4-aminobutyrate aminotransferase | ABAT | NM_001127448 | 16p13.2 | 10 |
|  | Acetylcholinesterase (Yt blood group) | ACHE | NM_015831 | 7q22 | 0^a^ |
| Signal transduction | Homer homolog 1 (Drosophila) | HOMER1 | NM_004272 | 5q14.2 | 3 |
|  | Citron (rho-interacting, serine/threonine kinase 21) | CIT | NM_007174 | 12q24 | 6 |
|  | Signal transducer and activator of transcription 4 | STAT4 | NM_003151 | 2q32.2-q32.3 | 4 |
|  |  | MPRIP | NM_015134 | 17p11.2 | 0^b^ |
|  | RAB1B, member RAS oncogene family | RAB1B | NM_030981 | 11q12 | 0^a^ |
| Synaptic proteins | Syntaxin2 | EPIM | NM_194356 | 12q24.33 | 2 |
|  | Syntaxin5A | STX5A | uc001nv1.2 | 11q12.3 | 0^a^ |
|  | Syntaxin 8 | STX8 | NM_004853 | 17p12 | 15 |
|  | Syntaxin 12 | STX12 | NM_177424 | 1p35.3 | 0^b^ |
|  | Synaptotagmin II | SYT2 | NM_177402 | 1q32.1 | 6 |
|  | Synaptotagmin IV | SYT4 | uc002law.2 | 18q12.3 | 0^b^ |
|  | Synaptotagmin IX | SYT9 | NM_175733 | 11p15.4 | 9 |
|  | Synaptotagmin VII | SYT7 | NM_004200 | 11q12-q13.1 | 0^b^ |
|  | Synaptotagmin VIII | SYT8 | NM_138567 | 11p15.5 | 0^a^ |
|  | Synaptojanin 1 | SYNJ1 | NM_003895 | 21q22.2 | 0^b^ |
|  | SV2 related protein homolog (rat) | SVOP | NM_018711 | 12q24.11 | 4 |
|  | Synuclein, gamma (breast cancer-specific protein 1) | SNCG | NM_003087 | 10q23.2-q23.3 | 0^b^ |
|  | Synaptosomal-associated protein, 25kDa | SNAP25 | NM_130811 | 20p12-p11.2 | 4 |
| Cell-cell interaction | Catenin (cadherin-associated protein), delta 2 (neural plakophilin-related arm-repeat protein) | CTNND2 | NM_001332 | 5p15.2 | 37 |
|  | Tubulin, beta | TUBB | NM_178014 | 6p21.33 | 0^b^ |
|  | Cortactin binding protein 2 | CTTNBP2 | NM_033427 | 7q31 | 5 |
|  | Plectin 1, intermediate filament binding protein 500kDa | PLEC1 | NM_201382 | 8q24 | 1 |
|  | Limbic system-associated membrane protein | LSAMP | NM_002338 | 3q13.2-q21 | 13 |
|  | Microtubule-associated protein 2 | MAP2 | NM_031845 | 2q34-q35 | 1 |
|  | Microtubule-associated protein 1B | MAP1B | NM_005909 | 5q13 | 1 |
|  | Neurexin 1 | NRXN1 | NM_001135659 | 2p16.3 | 40 |
|  | Intercellular adhesion molecule 1 (CD54), human rhinovirus receptor | ICAM1 | NM_000201 | 19p13.3-p13.2 | 0^b^ |
|  | Gap junction protein, alpha 1, 43kDa | GJA1 | NM_000165 | 6q21-q23.2 | 0^b^ |
|  | Contactin 1 | CNTN1 | NM_001843 | 12q11-q12 | 15 |
|  | Neurocan | NCAN | NM_004386 | 19p12 | 0^b^ |
|  | Malignancy-associated gene | MAG | NM_002361 | 19q13.1 | 0^b^ |
|  | Contactin 2 (axonal) | CNTN2 | NM_005076 | 1q32.1 | 1 |
|  | Nestin | NES | NM_006617 | 1q23.1 | 0^b^ |
|  | Carcinoembryonic antigen-related cell adhesion molecule 1 (biliary glycoprotein) | CEACAM1 | NM_001712 | 19q13.2 | 0^b^ |
|  |  | HTT | NM_0002111 | 4p16.3 | 5 |
| Apoptosis | BCL2-like 11 (apoptosis facilitator) | BCL2L11 | NM_0138621 | 2q13 | 3 |
|  | Caspase 1, apoptosis-related cysteine peptidase (interleukin 1, beta, convertase) | CASP1 | NM_002331 | 11q23 | 0^b^ |
|  | Caspase 6, apoptosis-related cysteine peptidase | CASP6 | NM_001226 | 4q25 | 0^b^ |
|  | Harakiri, BCL2 interacting protein (contains only BH3 domain) | HRK | NM_003806 | 12q24.22 | 0^b^ |
| Enzymes | Heme oxygenase (decycling) 1 | HMOX1 | NM_002133 | 22q13.1 | 3 |
|  | Tryptophan hydroxylase 1 (tryptophan 5-monooxygenase) | TPH1 | NM_4179 | 11p15.3-p14 | 2 |
|  | Superoxide dismutase 3, extracellular | SOD3 | NM_003102 | 4p15.3-p15.1 | 0^b^ |
| Transcription and translation regulation | Cyclin L1 | CCNL1 | NM_020307 | 3q25.31 | 0^a^ |
|  | Thyroid hormone receptor, alpha (erythroblastic leukemia viral (v-erb-a) oncogene homolog, avian) | THRA | NM_003250 | 17q11.2 | 0^b^ |
|  | Nuclear receptor subfamily 1, group H, member 2 | NR1H2 | NM_007121 | 19q13.3 | 0^b^ |
|  | Jun B proto-oncogene | JUNB | NM_00229 | 19p13.2 | 0^a^ |
|  | Retinoblastoma 1 (including osteosarcoma) | RB1 | NM_000321 | 13q14.2 | 0^b^ |

^a^ no intragenic SNPs on 6.0 SNP array

^b^  no SNP after quality control and LD pruning
